# Epigenetic silencing of long non-coding RNA *BM742401* in multiple myeloma: impact on prognosis and myeloma dissemination

**DOI:** 10.1186/s12935-020-01504-4

**Published:** 2020-08-25

**Authors:** Zhenhai Li, Shaji Kumar, Dong-Yan Jin, George A. Calin, Wee-Joo Chng, Kam-Leung Siu, Ming-Wai Poon, Chor Sang Chim

**Affiliations:** 1 Guangdong Key Laboratory of Genome Instability and Human Disease Prevention, Department of Biochemistry and Molecular Biology, Shenzhen University School of Medicine, Shenzhen, China; 2 Department of Medicine, Queen Mary Hospital, The University of Hong Kong, Pokfulam Road, Pokfulam, Hong Kong, China; 3 Division of Hematology, Mayo Clinic, Rochester, MN USA; 4 School of Biomedical Sciences, Queen Mary Hospital, The University of Hong Kong, Hong Kong, China; 5 Department of Experimental Therapeutics, The University of Texas MD Anderson Cancer Center, Houston, TX USA; 6 Department of Haematology-Oncology, National University Cancer Institute, Singapore, Singapore

**Keywords:** Multiple myeloma, *BM742401*, DNA methylation, Overall survival

## Abstract

**Background:**

Long non-coding RNA (lncRNA) *BM742401* is a tumor suppressor in gastric cancer and chronic lymphocytic leukemia. As the promoter and coding region of *BM742401* are fully embedded in a CpG island, we hypothesized that *BM742401* is a tumor suppressor lncRNA epigenetically silenced by promoter DNA methylation in multiple myeloma.

**Methods:**

Methylation-specific PCR and quantitative bisulfite pyrosequencing were performed to detect the methylation of *BM742401* in normal plasma cells, myeloma cell lines and primary myeloma samples. The expression of *BM742401* was measured by qRT-PCR. The function of *BM742401* in multiple myeloma cells was analyzed by lentivirus transduction followed by migration assay.

**Results:**

*BM742401* methylation was detected in 10 (66.7%) myeloma cell lines but not normal plasma cells, and inversely correlated with expression of *BM742401*. In primary samples, *BM742401* methylation was detected in 3 (12.5%) monoclonal gammopathy of undetermined significance, 9 (15.8%) myeloma at diagnosis and 8 (17.0%) myeloma at relapse/progression. Moreover, *BM742401* methylation at diagnosis was associated with inferior overall survival (median OS: 25 vs. 39 months; *P *= 0.0496). In myeloma cell line JJN-3, stable overexpression of *BM742401* by lentivirus transduction resulted in reduced cell migration (*P *= 0.0001) but not impacting cell death or proliferation.

**Conclusions:**

This is the first report of tumor-specific methylation-mediated silencing of *BM742401* in myeloma, which is likely an early event in myelomagenesis with adverse impact on overall survival. Moreover, *BM742401* is a tumor suppressor lncRNA by inhibiting myeloma cell migration, hence implicated in myeloma plasma cell homing, metastasis and disease progression.

## Background

Multiple myeloma is one form of hematological malignancy characterized by the accumulation and patchy infiltration of the bone marrow by neoplastic plasma cells, which accounts for approximately 10% of all hematologic malignancies [1]. Active multiple myeloma is characterized by ≥ 10% clonal plasma cells in the bone marrow in addition to the presence of end-organ damages, including hypercalcemia, renal failure, anemia, and/or lytic bone lesions, which are collectively known as CRAB features [2]. Multiple myeloma is often preceded by an entirely asymptomatic state, monoclonal gammopathy of undetermined significance (MGUS), that progresses into symptomatic myeloma at the rate of 1% per year [3]. Genetically, multiple myeloma is a heterogeneous disease with about half of the patients carrying non-hyperdiploid karyotypes (such as recurrent translocations involving immunoglobulin gene located at 14q32), whereas the other half carrying hyperdiploid karyotype (such as trisomies of odd number chromosomes) [4]. Despite major advances, multiple myeloma remains an incurable disease [5, 6].

Long non-coding RNA (lncRNA) is a novel class of RNA molecules of > 200 nucleotides in length without protein-coding capacity [7, 8]. Functionally, lncRNAs may regulate gene expression at both transcriptional and post-transcriptional levels, and hence are involved in multiple biological processes including development, differentiation or carcinogenesis [9, 10]. In particular, lncRNAs have been shown to be associated with the pathogenesis of multiple myeloma [11, 12]. For instance, lncRNA *CRNDE* (colorectal neoplasia differentially expressed) was found to be upregulated in primary myeloma samples and cell lines as compared with healthy controls, and associated with poor OS, and knockdown of *CRNDE* inhibited myeloma cell proliferation and colony formation and increased apoptosis and cell cycle arrest in G0/G1 phase [13], suggesting an oncogenic role for *CRNDE* in myeloma. On the other hand, knockdown of lncRNA *OIP5*-*AS1* has been shown to promote myeloma cell proliferation, cell cycle progression and inhibit apoptosis, suggesting *OIP5*-*AS1* is a tumor suppressor in myeloma [14].

DNA methylation is an epigenetic mechanism for gene regulation without alteration of the DNA sequence [15], which refers to the addition of a methyl (-CH_3_) group to carbon five position of the cytosine ring in a CpG dinucleotide catalyzed by DNA methyltransferases [16]. DNA regions enriched with CpG dinucleotides are called CpG islands [17, 18]. In the mammalian genome, promoter-associated CpG islands are localized to or in close proximity to the promoter region of more than half of the human genes [19], and involved in the regulation of gene expression by DNA methylation [20]. Aberrant promoter DNA methylation contributes to carcinogenesis including blood cancers [21]. In normal cells, majority of promoter-associated CpG islands are unmethylated, associated with a euchromatin configuration, and hence transcriptionally ready or active for gene expression [22]. In contrast, cancer cells are characterized by global DNA hypomethylation, and locus-specific hypermethylation of promoter-associated CpG islands of tumor-suppressor genes, resulting in downregulation, and hence loss of tumor suppressor functions [23–25]. For instance, long non-coding RNA KIAA0495 has been shown to be silenced by promoter DNA methylation in myeloma [26].

By RNA-seq, *BM742401*, localized to 18q11.2, was found to be downregulated in gastric cancer cells compared with normal tissues, which was associated with poor survival in patients with gastric cancer, and hence a potential tumor suppressor. Moreover, ectopic overexpression of *BM742401* inhibited gastric cancer metastasis through regulation of cell migration and invasion [27]. Recently, in chronic lymphocytic leukemia (CLL), *BM742401* was also found to be a tumor suppressor lncRNA, which was frequently methylated in primary samples of CLL [28]. As the promoter and coding region of *BM742401* are fully embedded in a CpG island, we hypothesized that *BM742401* may also be a tumor suppressor lncRNA epigenetically silenced by promoter DNA methylation in multiple myeloma. To verify this hypothesis, we studied the methylation status of *BM742401* promoter in healthy controls, myeloma cell lines and myeloma primary samples, and investigated its tumor suppressor function.

## Methods

### Patient information

Bone marrow samples were obtained from patients with MGUS (n = 24), newly diagnosed myeloma (n = 57) and myeloma relapse/progression (n = 47). Diagnosis of myeloma was based on standard criteria of the International Myeloma Working Group (IMWG) [29]. Complete staging work-up consisted of bone marrow examination, skeletal imaging, serum and urine protein electrophoresis, and/or serum free light chain levels. Of the 57 patients with newly diagnosed myeloma, there were 24 females and 33 males, with a median age of 71 (35–88) years. Apart from 11 patients lacking International Staging System (ISS) data [30], there were 10 stage I, 22 stage II, and 14 stage III cases. There were 12 IgA, 40 IgG, 4 light chain, and 1 non-secretary myelomas. According to the IMWG criteria, “relapse” was defined as the reappearance of the same paraprotein detected by serum/urine protein electrophoresis, appearance of new bone lesion or extramedullary plasmacytoma, or unexplained hypercalcemia after prior complete remission; while “progression” as increase of M-protein by 25% from lowest confirmed response value with an absolute rise of serum M-protein of ≥ 0.5 g/dL [31]. The study has been approved by the Institutional Review Board of Queen Mary Hospital (UW 05-269 T/932), and written informed consent was obtained from patient for publication of this article and any accompanying data or images. DNA of patient samples are extracted from bone marrow buffy coat, whereby malignant plasma cells are enriched by ficoll gradient centrifugation.

### Cell culture

Human myeloma cell lines (HMCLs) KMS-12-PE, MOLP-8, OPM-2 and U-266 were obtained from Deutsche Sammlung von Mikroorganismen und Zellkulturen (DSMZ, Braunschweig, Germany). NCI-H929 was purchased from American Type Culture Collection (ATCC, Manassas, VA, USA). KMS-11/BTZ and OPM-2/BTZ were acquired from Kyowa Hakko Kirin Co. Ltd. (Tokyo, Japan). LP-1 and RPMI-8226 were kindly provided by Prof. Robert Orlowski (Department of Lymphoma/Myeloma, Division of Cancer Medicine, The University of Texas MD Anderson Cancer Center, Houston, TX, USA). JJN-3, OCI-MY5 and RPMI-8226R were kindly provided by Prof. Wee Joo Chng (Department of Medicine, Yong Loo Lin School of Medicine, National University of Singapore). WL-2 was kindly provided by Prof. Andrew Zannettino (Myeloma Research Programme, The University of Adelaide, Australia). MMLAL [32] and MMKKF (unpublished) were established from the myelomatous pleural effusion of myeloma patients. Cell lines were cultured in RPMI-1640 medium (IMDM for LP-1, DMEM + IMDM for MMLAL), supplemented with 10% or 20% fetal bovine serum, 50 U/mL of penicillin and 50 μg/mL streptomycin, in a humidified atmosphere of 5% CO_2_ at 37 °C. All culture reagents were purchased from Invitrogen (Carlsbad, CA, USA).

### Methylation-specific polymerase chain reaction (MSP)

Detailed procedures of MSP have been previously described [33, 34]. Primer sequences and conditions are in Table 1.

**Table 1:** Primer sequences and PCR reaction conditions for *BM742401*

Primer set	Forward primer (5′–3′)	Reverse primer (5′–3′)	MgCl_2_/Tm/cycles	References
(I) *BM742401* MSP				
M-MSP	CGT TTA GGT AGA TAA TGA GAG TCG C	AAA TCA AAC GTT CTA TAA CCT CCG	1.5 mM/60 °C/38X	[28]
U-MSP	TGT GTT GTT TAG GTA GAT AAT GAG AGT TGT	CCA AAT CAA ACA TTC TAT AAC CTC CA	2.0 mM/60 °C/38X	
(II) qRT-PCR				
*BM742401*	TTG GTT CTT TTC TAC AAG GAT GTC	CGA ATC GGT CAA TGT CCA CC	NA	NA
*GAPDH*	ACC ACA GTC CAT GCC ATC ACT	TCC ACC ACC CTG TTG CTG TA	NA	NA

Tm, annealing temperature; M-MSP, methylated MSP; U-MSP, unmethylated MSP

### Quantitative bisulfite pyrosequencing

With bisulfite-treated DNA of HMCLs as template, specific PCR product overlapping the MSP amplicon was amplified by a pair of methylation-unbiased primers using PyroMark PCR Kit (Qiagen). Primer sequences are as followed: forward primer: 5′-AGG GGA GGA GAG AAA AGA G-3′; reverse primer: 5′-biotin–AAC TAT ACA CTA CCA ACT CCT-3′; condition: 2 mM MgCl_2_/61 °C/50 cycles. PCR product was purified and consecutive CpG dinucleotides was pyrosequenced with sequencing primer: 5′-GTT TAG GTA GAT AAT GAG AGT-3′ [28].

### Quantitative reverse transcription polymerase chain reaction (qRT-PCR)

Total RNA was isolated using mirVana™ miRNA Isolation Kit (Ambion, Austin, TX, USA). Reverse transcription was performed using QuantiTect Reverse Transcription Kit (Qiagen). *BM742401* was quantified using SYBR Green Master Mix (Applied Biosystems, Waltham, MA, USA) with *GAPDH* as endogenous control. Primer sequences of qRT-PCR for *BM742401* and *GAPDH* were listed in Table 1. Expression of *BM742401* was calculated by ∆CT method.

### 5-Aza-2′-deoxycytidine (5-AzadC) treatment

MOLP-8 cells, which were completely methylated for *BM742401*, were treated with 0.5 μmol/l, 1 μmol/l and 1.5 μmol/l 5-AzadC (Sigma-Aldrich, St. Louis, MO, USA) in fresh medium replaced every 24 h for 5 days. Cells were harvested for DNA and RNA extraction on day 5. Relative expression level of *BM742401* in 5-AzadC-treated group against untreated group was calculated by 2^−∆∆CT^ method.

### Lentivirus transduction

The full-length cDNA of *BM742401* was amplified and cloned into the XbaI and EcoRI sites of a pCDHCMV-MCS-EF1-copGFP lentivector (System Biosciences, Palo Alto, CA, USA; named empty vector) as described before [28], and the reconstructed vector was named *BM742401* vector. *BM742401* vector and empty vector were then co-transfected with pPACK packaging plasmid mix respectively into 293TN cells, followed by collection of supernatants at 48 h after transfection and concentration of pseudoviral particles by PEG-*it*™ Virus Precipitation Solution (System Biosciences). After pseudoviral titer estimation using 293TN cells, JJN-3 cells were transduced for 48 h by the pseudoviral particles with multiplicity of infection at 4. GFP-positive JJN-3 cells were selected by flow cytometry (BD FACSAria I Cell Sorter) and further cultured for 3 weeks. Relative expression of *BM742401* in response to transduction of *BM742401* vectors as compared with empty vectors was analyzed by 2^−∆∆CT^ method.

### Migration assay

To test the effect of *BM742401* overexpression on myeloma cell migration, we used bone marrow stromal cells (BMSCs) as a source for secreting chemoattractant for myeloma cells.

In the migration assay, a pilot transwell experiment was conducted to find out the optimal experimental conditions. At 24 h before migration assay, JJN-3 cells transduced with empty vector were starved by washing with PBS and resuspending in RPMI-1640 medium without FBS. The next day, in each of the transwell permeable support (8.0-μm polycarbonate membrane, 6.5-mm insert, and 24-well plate; Corning Costar, Tewksbury, MA, USA), 1 × 10^5^ starved JJN-3 cells were seeded in 200 μl RPMI-1640 medium. In the lower chamber, one of the following three conditions was used: (1) 500 μl RPMI-1640 medium with 20% FBS; (2) 500 μl BMSC conditioned medium (described below); or (3) 2 × 10^5^ BMSCs in 500 μl DMEM medium with 10% FBS that had been seeded on the day before. BMSCs were cultured from normal bone marrow donors as previously described [35]. “BMSC conditioned medium” was generated by mixing the filtered culture medium of BMSCs (at 37 °C in 5% CO_2_ for 24 h) with 20% FBS in RPMI-1640 medium at the ratio of 1:1. After 72 h of incubation at 37 °C, the GFP-positive cells that had migrated to the lower chambers were counted using fluorescence microscope (Axiovert 135, ZEISS microscopy, Germany). The rationale of the use of BMSCs in the lower chamber stemmed from the concept that myeloma cell homing is mediated by SDF-1 produced by BMSCs that bind to the CXCR4 receptor on myeloma cells, hence myeloma cells would migrate to the bone marrow niches due to this concentration gradient [36]. As the highest myeloma cell migration occurred with BMSCs laid at the bottom of the lower chamber (Additional file 1: Figure S1), which hence was adopted for subsequent transwell experiments to compare migration efficiency between JJN-3 cells transduced with empty vector and *BM742401* vector. Triplicate experiments were performed for each group, and the means and standard deviations were calculated.

### Trypan blue exclusion assay

Cell death was analyzed by trypan blue (Sigma-Aldrich) at day 3 after seeding cells. Cells in five random microscopic fields were counted for each group under microscope. Dead cell (stained in blue) percentage = average number of dead cells per microscopic field/average number of total cells per microscopic field.

### MTS assay

The number of viable cells in proliferation was measure by CellTiter 96^®^ AQ_ueous_ One Solution Cell Proliferation Assay Kit (Promega, Madison, WI, USA) following the manufacturers’ instructions. Relative proliferation percentage of *BM742401* overexpressed cells compared with control cells was calculated at day 5 after seeding cells.

### Statistical analysis

Overall survival (OS) was measured from the date of diagnosis to the date of last follow-up or death. OS of patients with and without *BM742401* methylation were compared. Survival was plotted by the Kaplan–Meier method, and compared by the log-rank test. The difference between JJN-3 cells transduced with *BM742401* vectors and empty vectors in migration assay was studied by Student’s *t* test. All *P* values were two-sided and *P* < 0.05 was defined as significant difference.

## Results

### Methylation of *BM742401* in healthy controls and human myeloma cell lines (HMCLs)

**Fig. 1:**
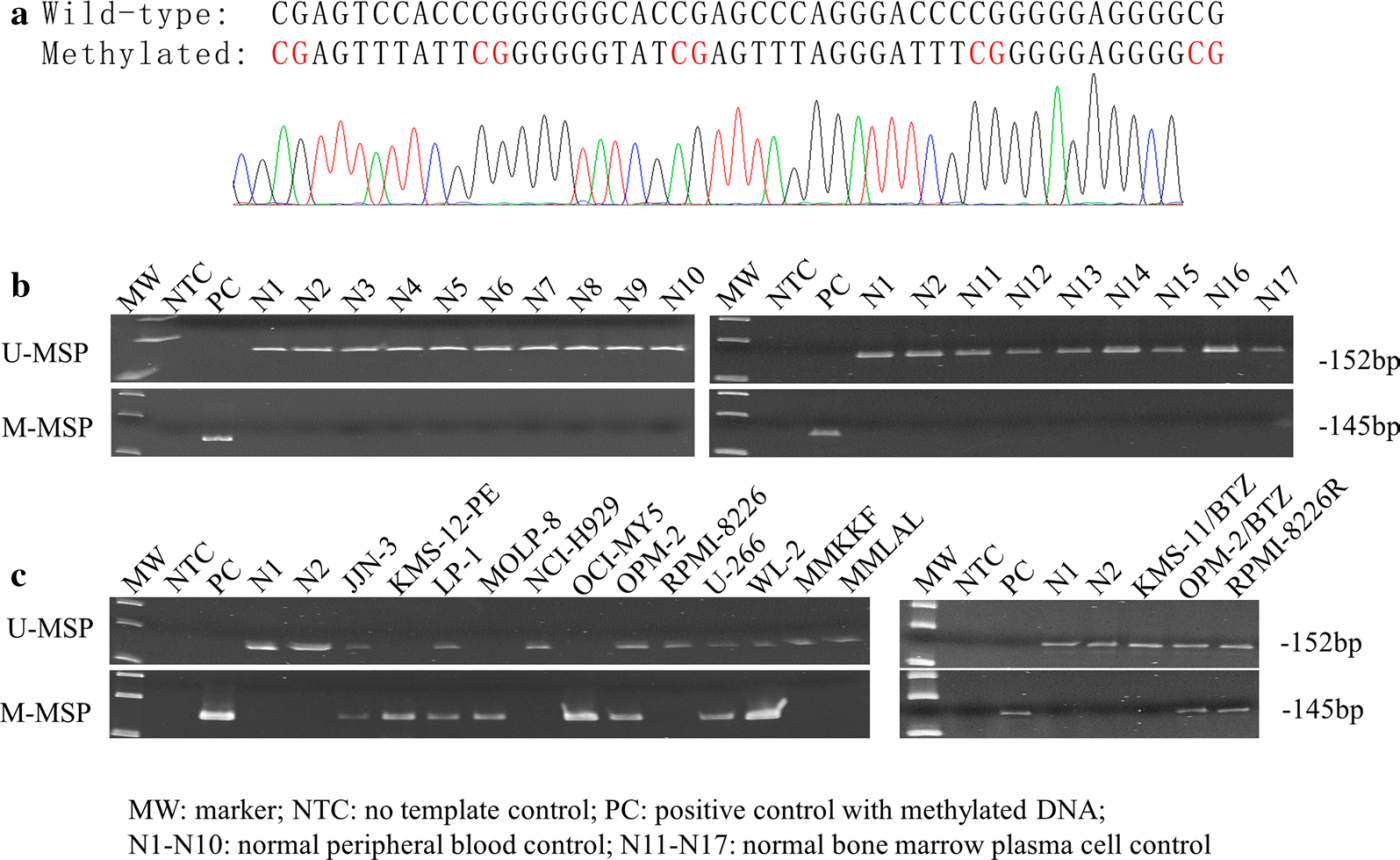
Methylation of *BM742401* in healthy controls and HMCLs. **a** Direct sequencing of M-MSP products from positive control with methylated DNA showed the conversion of all unmethylated cytosines into uracils (turned into thymidines after PCR) but all methylated cytosines remained unchanged, indicating complete bisulfite conversion and specificity of MSP. **b** M-MSP and U-MSP showed that all healthy controls (N1-N17) were completely unmethylated (UU), whereas positive control with methylated DNA was completely methylated (MM). **c** M-MSP and U-MSP showed *BM742401* was MM in HMCLs, including KMS-12-PE, MOLP-8 and OCI-MY5, partially methylated (MU) in JJN-3, LP-1, OPM-2, U-266, WL-2, OPM-2/BTZ and RPMI-8226R, UU in NCI-H929, RPMI-8226, MMKKF, MMLAL and KMS-11/BTZ

MSP was carried out to examine methylation of *BM742401* in the bisulfite-converted DNA of healthy controls [peripheral blood (n = 10) and CD138-sorted bone marrow plasma cell (n = 7)] and HMCLs (n = 15). Direct sequencing of the M-MSP products from positive control with methylated DNA confirmed complete bisulfite conversion and MSP specificity, as indicated by conversion of all unmethylated cytosines into thymidines after PCR, whereas all methylated cytosines remained unchanged (Fig. 1a). None of the healthy controls showed methylation of *BM742401* (Fig. 1b). By contrast, in HMCLs, *BM742401* was completely methylated (MM; M-MSP positive but U-MSP negative) in KMS-12-PE, MOLP-8 and OCI-MY5, partially methylated (MU; both M-MSP and U-MSP positive) in JJN-3, LP-1, OPM-2, U-266, WL-2, OPM-2/BTZ and RPMI-8226R, and completely unmethylated (UU; M-MSP negative but U-MSP positive) in NCI-H929, RPMI-8226, MMKKF, MMLAL and KMS-11/BTZ (Fig. 1c). Moreover, these MSP methylation statuses (MM, MU, and UU) were verified using quantitative bisulfite pyrosequencing, which showed that completely methylated HMCLs were associated with a higher methylation level between 63.4% to 85.4%, partially methylated HMCLs carried an intermediate methylation level of 36.9% to 49.6%, and completely unmethylated HMCLs were associated with a lower methylation level from 15.1% to 23.6% (Additional file 2: Figure S2). These data suggested that *BM742401* was methylated in a tumor-specific manner in myeloma.

### Methylation and expression of *BM742401* in HMCLs

**Fig. 2:**
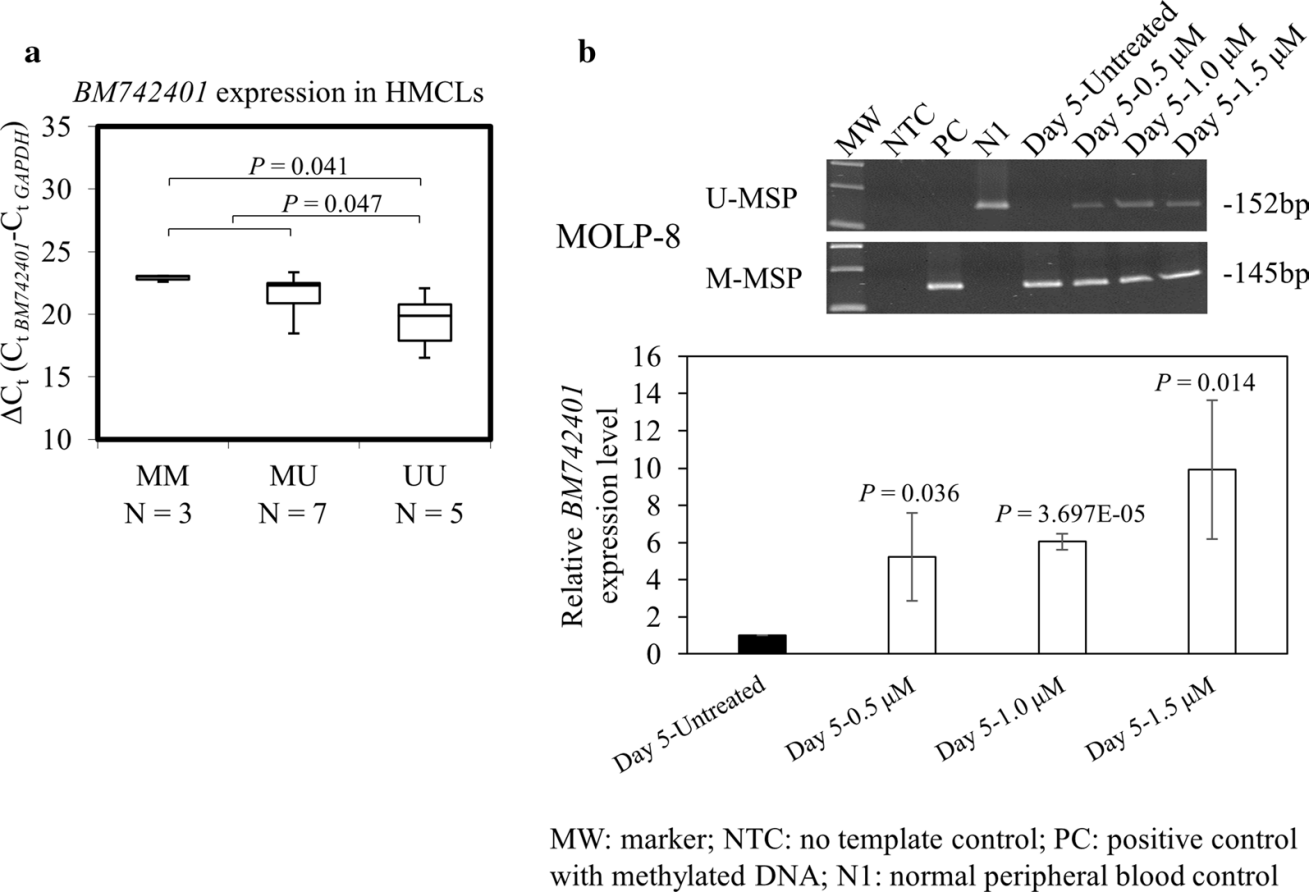
Methylation and expression of *BM742401* in HMCLs. **a** By qRT-PCR, methylation of *BM742401* was significantly correlated with lower expression level and hence larger ΔCt of *BM742401* (MM vs. UU, *P* = 0.041; MM + MU vs. UU, *P* = 0.047). **b** In MOLP-8 cells, which were completely methylated for *BM742401*, treatment with 5-AzadC for 5 days led to *BM742401* promoter demethylation, as evidenced by emergence of U-MSP signal (upper), and concomitant re-expression of *BM742401* (lower). Error bars represent standard deviation from three independent qRT-PCR

To study if methylation was correlated with repression of *BM742401*, qRT-PCR was employed to measure the expression levels of *BM742401* in HMCLs. Results showed that HMCLs with methylation of *BM742401* had significantly lower expression levels of *BM742401* (Fig. 2a; MM vs. UU, *P* = 0.041; MM + MU vs. UU, *P* = 0.047) than HMCLs that were completely unmethylated.

To further testify if promoter DNA methylation resulted in downregulation of *BM742401*, MOLP-8 cells, which were completely methylated for *BM742401*, were treated with 5-AzadC, a demethylation agent. Upon treatment with 5-AzadC, the promoter of *BM742401* was demethylated as evidenced by the emergence of U-MSP signal on day 5 (Fig. 2b). Moreover, by qRT-PCR, *BM742401* was simultaneously re-expressed by 5.2 to 9.9 folds with different concentrations of 5-AzadC (Fig. 2b). Therefore, in myeloma cells, methylation-mediated silencing of *BM742401* was reversible.

### Methylation of *BM742401* in primary bone marrow samples

**Fig. 3:**
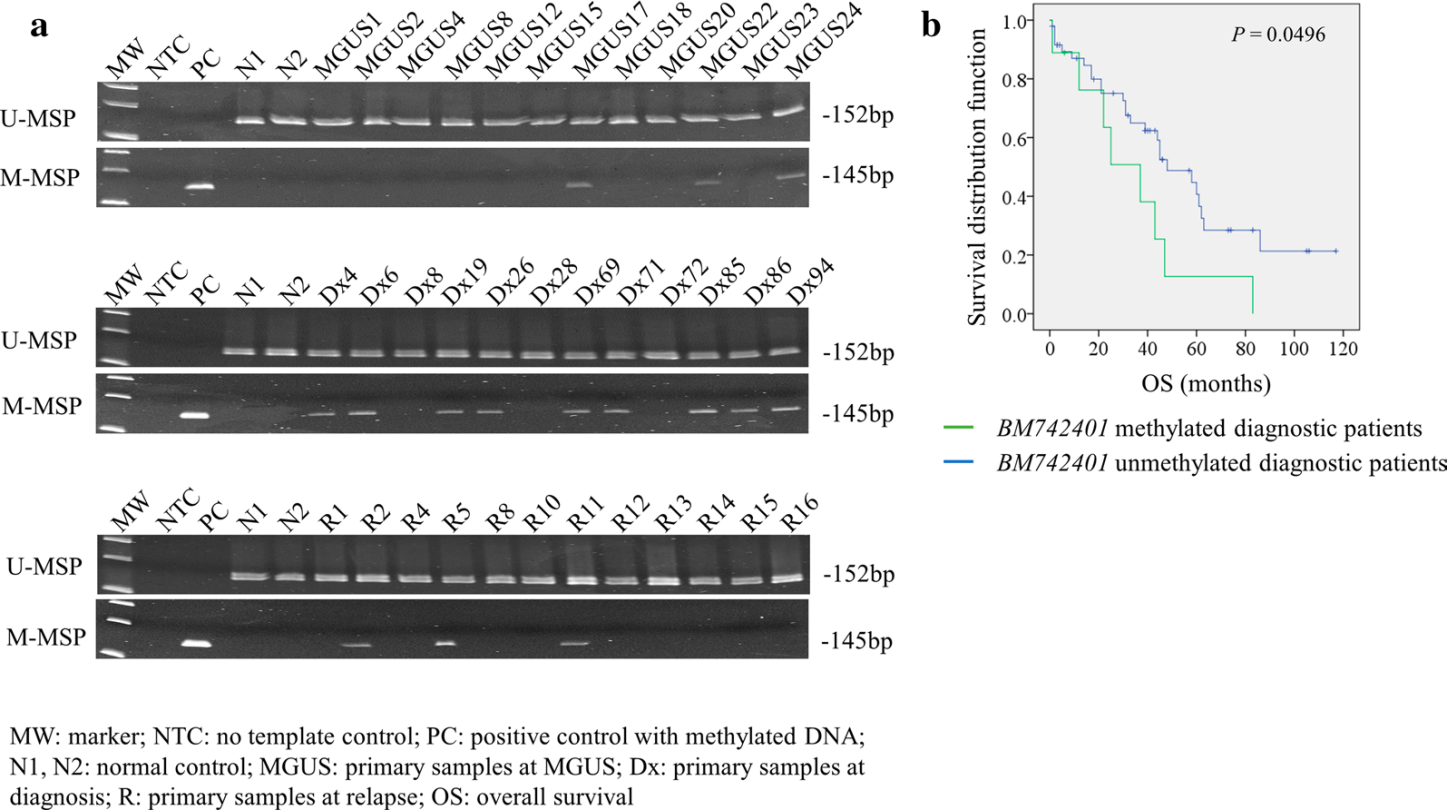
Methylation and expression of *BM742401* in primary bone marrow samples. **a** Representative M-MSP and U-MSP showing methylation of *BM742401* in primary samples of MGUS (total: n = 24), myeloma at diagnosis (total: n = 57) and myeloma at relapse/progression (total: n = 47). The numbers were assigned for illustration purpose, and hence, the identical Arabic numerals in different disease stages are not serial samples from the same patient. **b** Kaplan–Meier analysis of OS in patients with and without methylation of *BM742401*

By MSP, methylation of *BM742401* was detected in primary bone marrow samples of 3 (12.5%) MGUS, 9 (15.8%) myeloma at diagnosis, and 8 (17.0%) myeloma at relapse/progression (Fig. 3a). Methylation frequency of *BM742401* was not significantly different among those consecutive clinical stages of myeloma (MGUS vs. myeloma at diagnosis: *P* = 1.000; myeloma at diagnosis vs. myeloma at relapse/progression: *P* = 1.000). In contrast to absence of methylation in normal, presence of methylation in MGUS with a frequency comparable to consecutive stages from MGUS to myeloma at diagnosis and relapse/progression indicated *BM742401* methylation might be an early event in the pathogenesis of myeloma.

Interestingly, by Kaplan–Meier analysis, the projected overall survival (OS) of diagnostic myeloma patients with and without *BM742401* methylation were 11.1% and 45.8% respectively, and patients with *BM742401* methylation (n = 9) showed significantly shorter OS than patients without *BM742401* methylation (n = 48; Fig. 3b; median OS: 25 vs. 39 months; *P *=0.0496).

### Tumor suppressive function of *BM742401* in myeloma cells

**Fig. 4:**
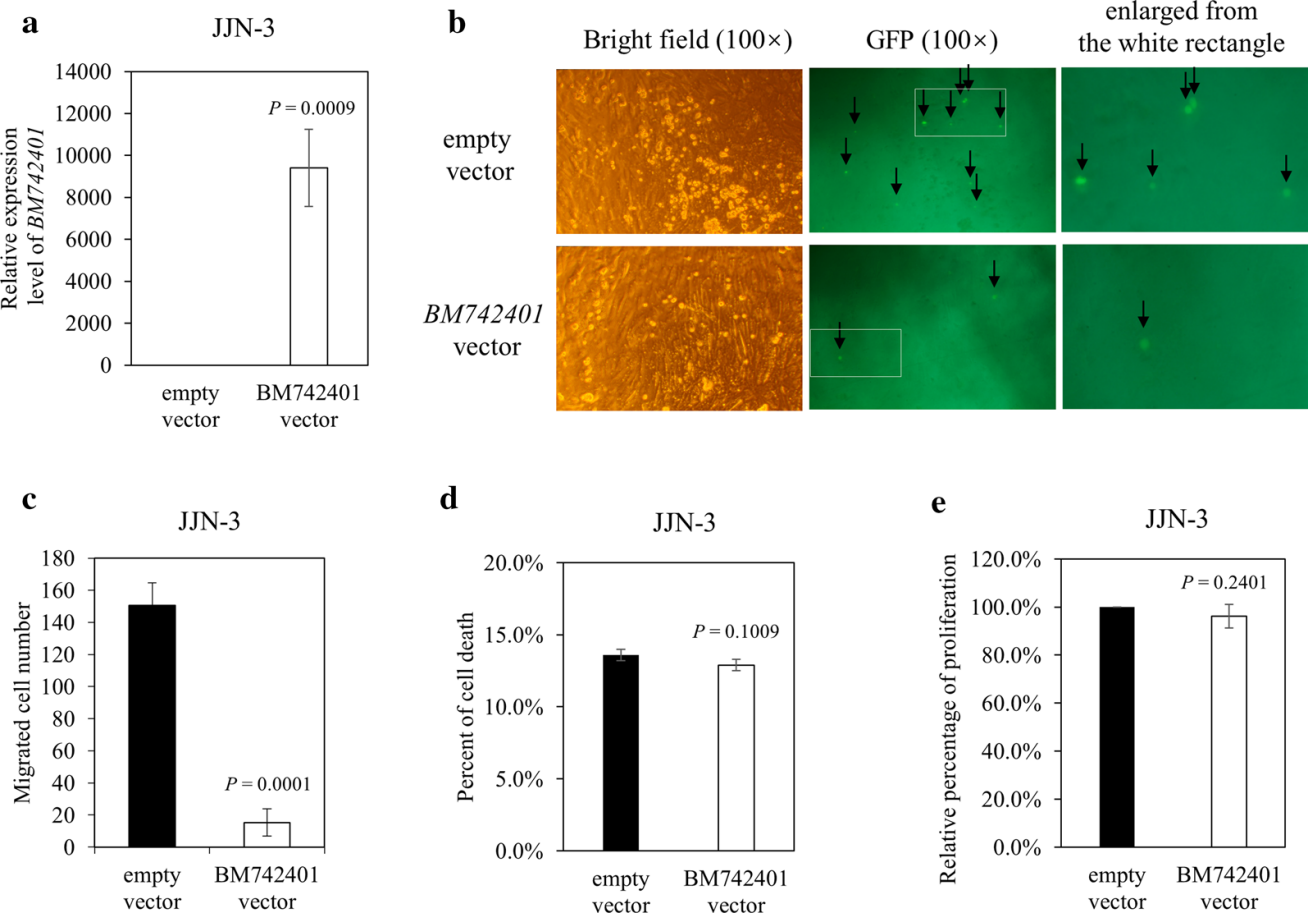
Function of *BM742401* in HMCL. **a** By qRT-PCR, *BM742401* was shown to be successfully overexpressed in JJN-3 cells. **b** Representative GFP and bright field images of JJN-3 cells that migrated into the lower chambers are shown (Each arrow points to one GFP positive cell). **c** At 72 h after the transduced JJN-3 cells were seeded, GFP-positive cells that migrated to the lower chambers were counted using fluorescence microscope. (**d**, **e**) Trypan blue exclusion assay (**d**) and MTS assay (**e**) of JJN-3. Error bars represent standard deviation from three independent experiments

As *BM742401* was frequently methylated in HMCLs and primary samples, we postulated that it might act as a tumor suppressor. By lentivirus transduction, *BM742401* was stably overexpressed by 9397.0 folds in JJN-3 cells compared with empty vector control (Fig. 4a and Additional file 3: Figure S3; *P* = 0.0009). Moreover, overexpression of *BM742401* resulted in reduced cell migration of JJN-3 cells by transwell migration assay (Fig. 4b and c; *P* = 0.0001), but not affecting cell death (Fig. 4d; *P* = 0.1009) or proliferation (Fig. 4e; *P* = 0.2401) by trypan blue exclusion assay and MTS assay respectively. Therefore, *BM742401* exhibits its tumor suppressor property in myeloma by inhibiting cell migration.

## Discussion

### There are a number of interesting observations in this study

Firstly, methylation of *BM742401* was tumor-specific as it was absent in normal controls, whereas frequently detected in HMCLs and primary myeloma samples, which is similar to the tumor-specific methylation of other tumor suppressive protein coding genes [37, 38], miRNAs [39, 40] and lncRNA [26] in myeloma. In contrast, methylation of some miRNAs, such as miR-9-2 and miR-373 [41, 42], occurred in both cancer cells and their normal counterparts, and hence methylated in a tissue-specific manner, thereby unimportant in carcinogenesis.

Secondly, methylation-mediated silencing of *BM742401* was shown to be reversed by treatment of demethylating agent, consistent with the reversible silencing of *BM742401* shown in CLL [28], indicating that promoter DNA methylation is also a mechanism for repression of tumor suppressor lncRNAs in myeloma.

Thirdly, in primary samples, methylation of *BM742401* appeared as early as MGUS, at a frequency comparable to that of active myeloma at diagnosis and relapse/progression. Therefore, it is likely that methylation of *BM742401* is an early event in the pathogenesis of myeloma, similar to methylation of *miR*-*203* [40] and *miR*-*342* [34]. By contrast, *miR*-*129*-*2* methylation was implicated in the progression from MGUS to symptomatic myeloma [43], and *miR*-*34b/c* methylation at relapse/progression of myeloma [39]. Moreover, methylation of *BM742401* correlated with shorter OS in newly diagnosed myeloma, similar to *CDKN2A* [44, 45] and *DAPK1* [46] methylation, suggesting an adverse impact of *BM742401* methylation for OS. As this cohort of myeloma patients is small and not uniformly treated, the prognostic significance of *BM742401* methylation needs to be verified by multivariate analysis in a larger cohort of uniformly-treated patients.

Fourthly, the tumor suppressor function of *BM742401* has been shown in gastric cancer [27] and CLL [28]. Herein, we confirmed *BM742401* as a tumor suppressor in myeloma, as evidenced by the inhibition of myeloma cell migration in myeloma cells with stable overexpression of *BM742401*. In myelomagenesis, there is constant trafficking of myeloma cells through the blood to the bone marrow niches, a process termed homing, due to secretion of SDF-1 by BMSC, thereby creating a concentration gradient from the BM stroma to the circulating plasma cells [47]. Indeed, in our pilot migration experiment using one of the following three conditions in the lower chamber including FBS only, BMSC conditioned medium, or BMSCs, lower chamber loaded with BMSCs resulted in the highest yield of myeloma cell migration across the membrane (Additional file 1: Figure S1), implicating the importance of the SDF-1/CXCR4 axis in plasma cell migration [36]. Indeed, myeloma cells with stable overexpression of *BM742401* led to significant inhibition of myeloma plasma cell migration than cells with empty vector using this condition. Therefore, epigenetic silencing of *BM742401* may enhance myeloma metastasis and disease progression. This is consistent with the adverse impact of *BM742401* methylation on OS in our cohort, hence warrants further investigation.

By contrast, overexpression of *BM742401* did not influence myeloma cell death or proliferation, hence similar to the findings in gastric cancer that *BM742401* inhibited gastric cancer cell migration and invasion but not cell viability [27], but different from CLL in that *BM742401* inhibited CLL cell proliferation and enhanced apoptosis [28]. Therefore, the tumor suppressor function of *BM742401* appears cancer-type specific.

Lastly, *BM742401* localizes in an antisense direction to a neighboring protein-coding gene *GATA6*. As lncRNA may involve in the regulation of its neighboring gene, methylation of *BM742401* and expression of *GATA6* may be studied in myeloma. For example, *HOTTIP* expression was correlated to the activation of HOX genes, including *HOXA7*, *9*, *10*, *11*, and *13*, in the HOXA locus [9]. Moreover, *GATA6* may regulate the WNT signaling pathway, which is dysregulated in myeloma [48, 49], thereby methylation of *BM742401* may link to the regulation of the Wnt signaling pathway, playing a role in the pathogenesis of myeloma.

## Conclusions

In myeloma, methylation-mediated silencing of *BM742401* is tumor-specific, reversible, associated with inferior OS, and likely an early event in myelomagenesis, and *BM742401* is a tumor suppressor lncRNA by inhibiting myeloma cell migration, hence implicated in myeloma plasma cell homing and metastasis.

## Supplementary information

**Additional file 1: Figure S1.** Pilot transwell migration assay. JJN-3 cells transduced with empty vector were starved and seeded on the transwell support.

**Additional file 2: Figure S2.**Quantitative bisulfite pyrosequencing analysis of *BM742401* in HMCLs.

**Additional file 3: Figure S3.**Representative GFP (upper) and bright field images (lower) of JJN-3 cells stably transduced with empty vector or B*M742401* vector.

## Data Availability

All data generated or analyzed during this study are included in this published article and its Additional files 1, 2, 3.

## Abbreviations

lncRNA: Long non-coding RNA
MGUS: Monoclonal gammopathy of undetermined significance
miRNA: microRNA
MSP: Methylation-specific PCR
HMCLs: Human myeloma cell lines
M-MSP: Methylated MSP
U-MSP: Unmethylated MSP
MM: Completely methylated
MU: Partially methylated
UU: Completely unmethylated
PCR: Polymerase chain reaction
qRT-PCR: Quantitative real-time PCR
5-AzadC: 5-Aza-2’-deoxycytidine
OS: Overall survival
