# Supplementary figures and images for: Epigenetic silencing of long non-coding RNA BM742401 in multiple myeloma: impact on prognosis and myeloma dissemination
Source: Cancer Cell Int. 2020 Aug 25;20:403. doi: 10.1186/s12935-020-01504-4 (PMC7446116; doi:10.1186/s12935-020-01504-4)

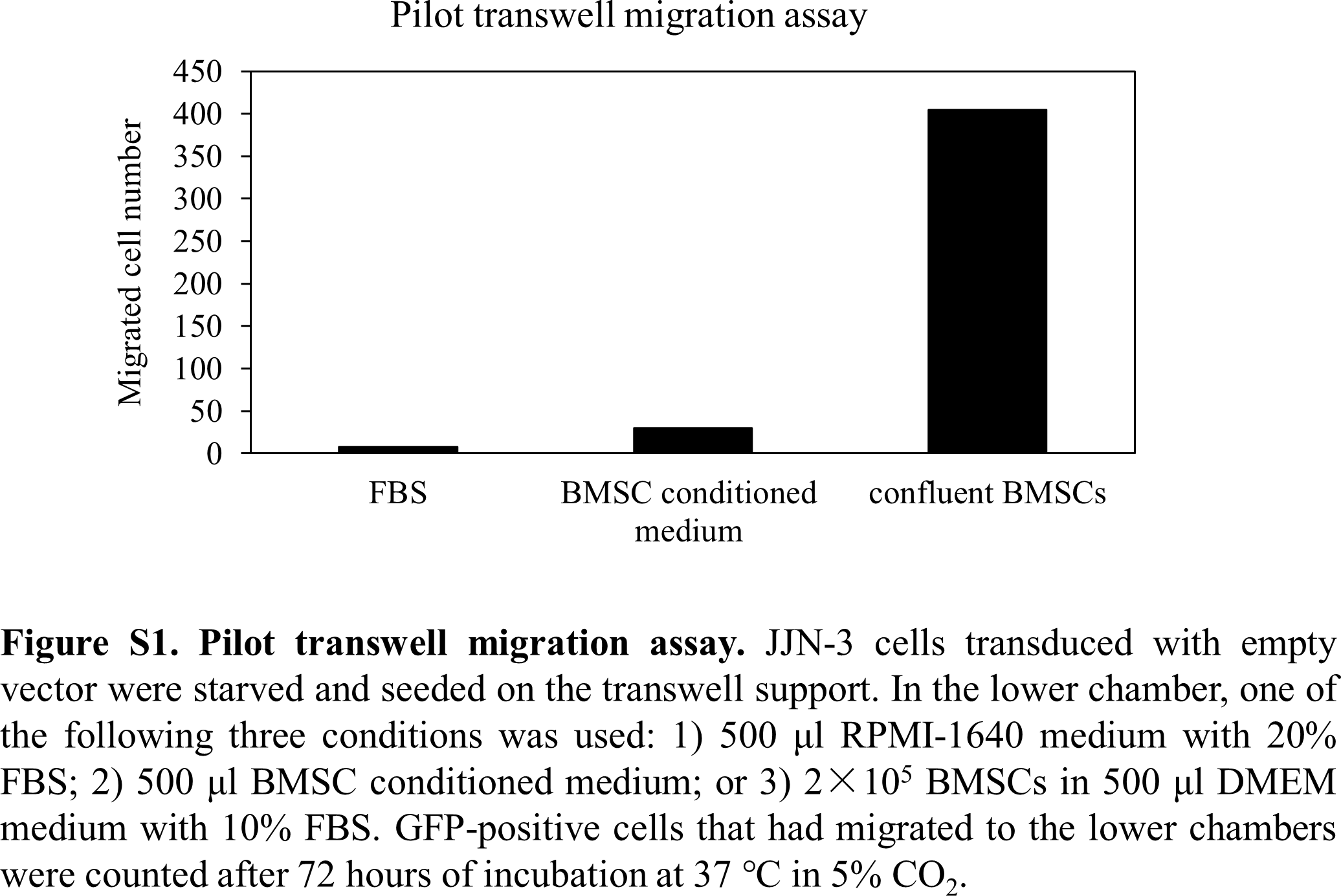

Supplement: Supplementary file 1 — Additional file 1: Figure S1. Pilot transwell migration assay. JJN-3 cells transduced with empty vector were starved and seeded on the transwell support. [file 12935_2020_1504_MOESM1_ESM.tif]

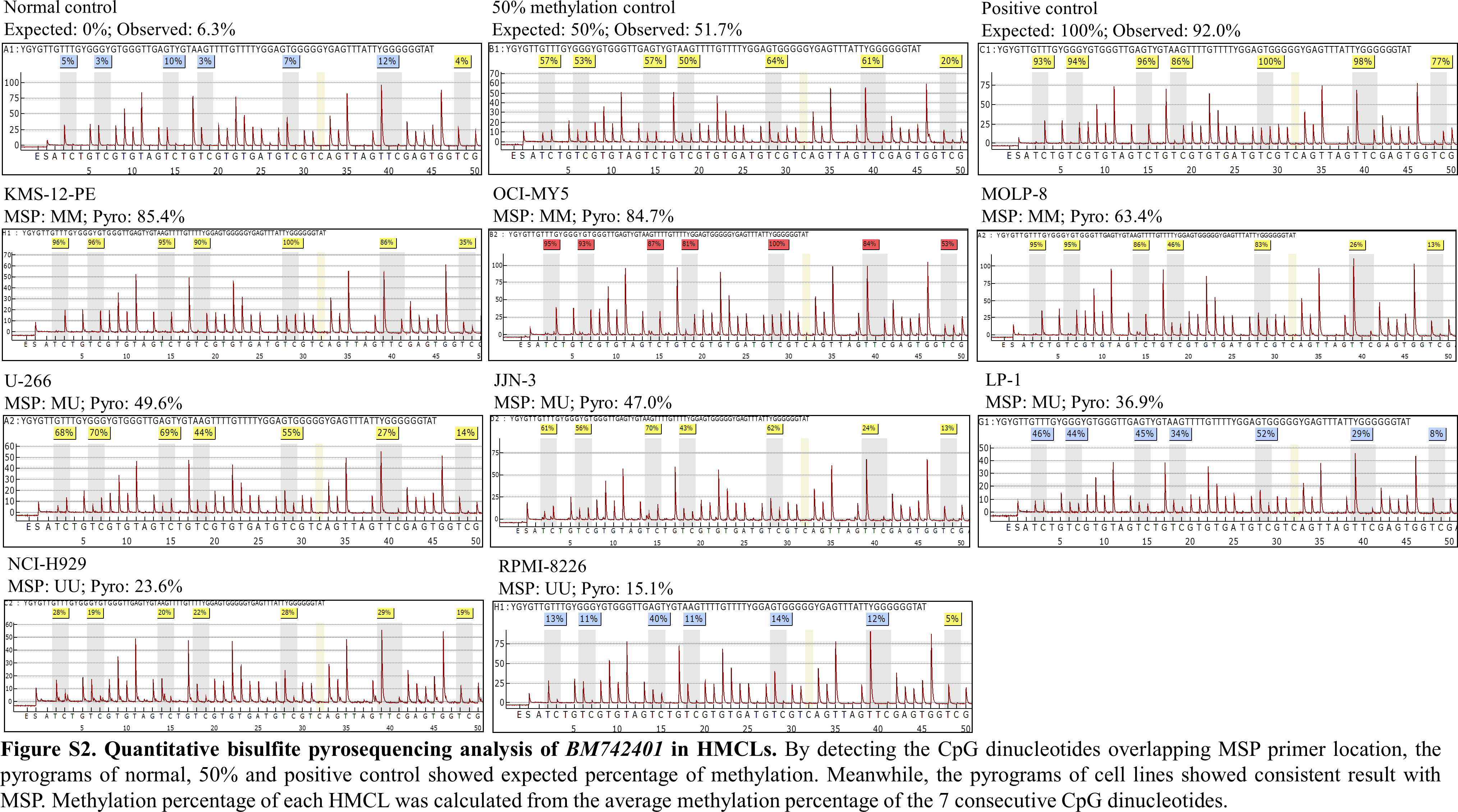

Supplement: Supplementary file 2 — Additional file 2: Figure S2.Quantitative bisulfite pyrosequencing analysis of BM742401 in HMCLs. [file 12935_2020_1504_MOESM2_ESM.tif]

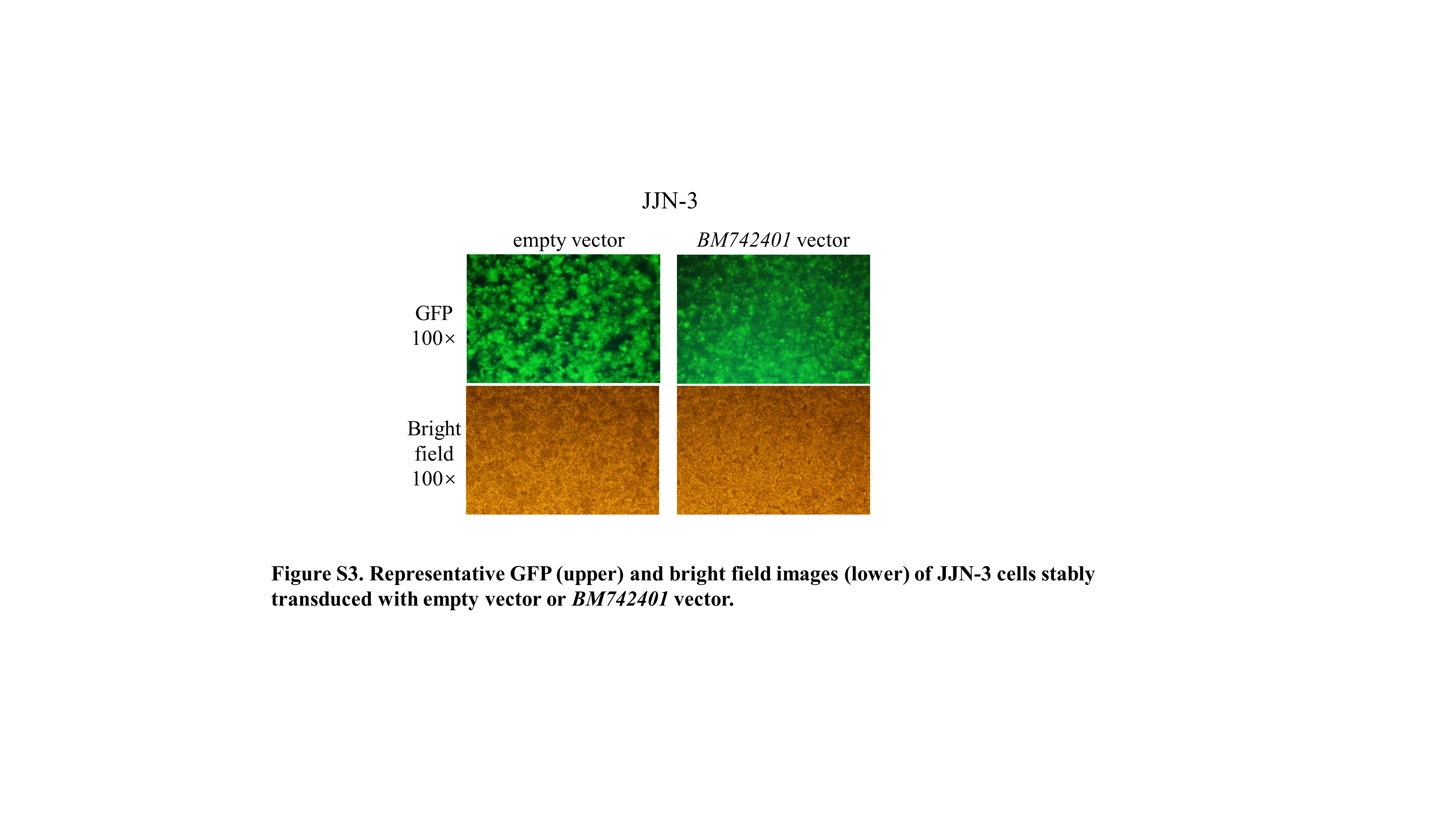

Supplement: Supplementary file 3 — Additional file 3: Figure S3.Representative GFP (upper) and bright field images (lower) of JJN-3 cells stably transduced with empty vector or BM742401 vector. [file 12935_2020_1504_MOESM3_ESM.tif]
